# Efficacy of Life Skills Training on Subjective Well-Being of Students: A Report from Rafsanjan, Iran

**Published:** 2014

**Authors:** Rezvan Sadr-Mohammadi, Mehrdad Kalantari, Hossein Molavi

**Affiliations:** 1 Department of Psychology, Kar Higher Education Institute of Rafsanjan, Rafsanjan, Iran.; 2 Associate Professor, Department of Clinical Psychology, School of Psychologic and Education Science, Isfahan University, Isfahan, Iran.

**Keywords:** Female, Life Skills Training, Students, Subjective Well-Being

## Abstract

**Objective:** The aim was to investigate the efficacy of life skills training on subjective well-being (SWB) among high school females.

**Methods:** The population study comprised all female high school of Rafsanjan, Iran, in 2008-2009. Thirty students with the lowest scores according to the Molavi’s SWB questionnaire were considered eligible. At the next stage, the required sample of 30 students were selected randomly and divided into two groups of experimental (15 subjects) and control (15 subjects). Then, life skills training sessions were started for the experimental group (eight sessions in a 4-week period). Control group did not receive any intervention. The method of data processing at a descriptive level was through using central tendency indicators, dispersion, frequency, and percentage. Student’s t-test was used for analysis of independent variables.

**Results: **The greatest R^2^ (0.48) was observed for SWB. The R^2^ coefficients for neurosis, stress-depression, vitality, and life determination were 0.27, 0.15, 0.20, and 0.09, respectively.

**Conclusion:** Life skills training showed the greatest effect regarding SWB of the students.

## Introduction

The changes appeared in human life have caused more psychological disorders than in the past (1).

In recent years, we have seen a trend in the psychological literature that focus on bad efficiency and disruption has changed to focus on positive mental health and well-being. This positive observation has been accepted in the World Health Organization (WHO) and health is defined as social, psychological, and physical well-being and not merely absence of illness (2).

The subjective well-being (SWB) concept relates to the subjective appreciation of life by the individual, an internal experience, apart from external criteria. SWB points to person’s assessment of his/her life (3). SWB is defined as a multifaceted concept including a cognitive and an affective dimension. The concept has also been defined through three basic components: satisfaction with life, positive affect, and negative affect (4). Joseph Sirgy described the three components as: (A) experience of “positive affect” in important domains of life; (B) the experience of “negative affect” in important domains of life; and (C) the evaluation of “satisfaction with life” as a whole or in several domains, but the topic with a few attention, does not mean that people in all of their life time have a good feeling (5). In addition, experience of painful excitement is a part of life, but the long-term ability to manage these painful and negative effects for well-being is needed, and life skills training is a method to achieve this goal.

Life skills include group of skills and abilities, which help individual for efficient resistance and also in attending to life situations and conflictions. According to the WHO 10 essential life skills are the ability to have effective communication, the ability for effective interpersonal relationship, decision-making ability, problem solving ability, creative thinking ability, critically thinking ability, the ability of being aware of the self, the ability of having sympathy with others, the ability to deal with emotions (failure, anxiety, depression, etc.), and the ability to deal with stress. These skills enable individuals to act in an adaptive way related to the environment and providing well-being.

An important point is that all life skills are attainable. These skills help individual in controlling problems such as depression, anxiety, rejection, diffidence, anger, and confliction in interpersonal relationship (6). Several studies have attended to these problems. For example, a study which consisted of 500 students showed that life skills’ training is effective in increasing mental and physical health and also in decreasing behavioral and social problems (7).

It has been shown that providing coping skills for students leads to improvement of performance and growth of quality of life in those with severe mental illness (8). Weitlauf et al. in a study on women under the title of conveying the effect of coping skills training on the self-assertiveness and aggression showed that such training caused the growth of assertiveness and self-efficacy of individuals (9). Also, in terms of interpersonal relationship, signification reduction was found at the levels of aggression and hostility.

Smith et al. study showed that life skills training significantly decreased alcohol consumption and drug abuse in the youth (6). Sukhodolsky et al. showed that the training of coping skills caused improvement of interpersonal relationship and the reduction of aggression and behavioral problems (10). Smith showed that life skills training had a significant effect on management and leadership abilities of young people (6). Mishara and Ystgaard showed that students following coping skills with stress tended to be more satisfied and reported less study-related mental pressure (11). Matsuda and Uchiyama showed that providing copping skills with stressful situations resulted in prevention and reduction of mental disorders and psychosomatic diseases in many people (12).

Adolescence is recognized as a particularly stressful period of development. During this period adolescents simultaneously deal with physical and cognitive transformations and need adaption. Therefore, because the majority of students have access to life skills instruction in their schools, this study can be implemented as a universal one which its consequences will be the improvement of SWB of female students of high schools in Rafsanjan, Iran. The main question of the study was that to which variable under investigation the study of life skills has more effect?

## Materials and Methods

The method of the study was experimental with pre- and post-tests and control group. The study population consisted of all female students in high schools of Rafsanjan, Iran in 2008-2009. To limit the probable effect of gender on the outcomes of the study, only female students were included. For selecting sample, random sampling was conducted at two stages. At the first stage, a female high school was selected randomly. Then, the SWB questionnaires were administrated for 148 students. At the next stage, the students with the lowest scores (i.e., 30 subjects) were selected through using simple random sampling and were replaced in experimental (15 subjects) or control (15 subjects) groups.

A series of etiologic studies examining mediating mechanisms has been conducted by our research group at Cornell University in an attempt to explore the roles of social influences, psychosocial characteristics, and social and personal competence skills in the etiology of depression, anxiety, and happiness in life. Three findings that have emerged from our research are that competence skills are necessary for present and future females: (a) by increasing psychological well-being, (b) by reducing conflicts with parents and friends and improving positive interpersonal relations, and (c) by increasing assertiveness and ability of stress management.

Life skills trainings were instructed in eight sessions by the researchers, two sessions each week.

For data collecting, the Molavi’s questionnaire of neurosis, stress and depression, vitality, and life determination which contains 39 items and is used for measuring general subjective well-being was applied (13). This questionnaire is a combination of Diener positive and negative affect, Andrews and Withney life satisfaction, Myers’ vitality, and Tsaousis optimism-pessimism questionnaires. The Cronbach’s alpha coefficients for this scale on 0.7 norms were as follows: neurosis 0.8, stress-depression 0.73, vitality 0.92, and life determinate 0.89. Extensive evidence exists for concurrent validity of this questionnaire. In brief, correlations between the negative affect score and measures of depression and anxiety typically range from 0.60 to 0.80 and in test-retest method for these scales are 0.53-0.78 respectively. Also, a demographic questionnaire was used for gathering data about economic, social, personal, and family aspects. The Ethics Committee of Esfahan University approved the study.

The method of data processing at a descriptive level was through using central tendency indicators and dispersion and frequency and percentage. For analytical analyses, the Student’s t-test was used for independent variables.

## Results

Table 1 presents demographic variables. The SWB showed the highest change among other variables. Also, the parameter R^2^ was 0.48 for SWB. The R^2^ coefficients for neurosis, stress-depression, vitality, and life determination were 0.27, 0.15, 0.20, and 0.09, respectively (Table 2).

In table 3, mean difference of experimental scores and control group on SWB has been compared. The calculated t parameter shows that the difference between average scores of SWB between experimental and control group was significant (p < 0.01).

**Table 1:** Demographic variables of the studied sample

**Variables**	**Experimental group**	**Control group**
**Age**		
**Mean**	15.6	15.4
**SD** †	1.76	2.13
**Parental education**		
**Below junior level**		
**Father**	26.7	53.3
**Mother**	46.7	60.0
**High school**		
**Father**	46.7	26.7
**Mother**	33.3	26.7
**4-year college**		
**Father**	26.7	30.0
**Mother**	30.0	13.3
**Master or doctorate**		
**Number of family members**		
**Mean**	8	6
**SD**	1.87	2.50
**School final score average**		
**Mean**	15.83	15.20
**SD**	3.78	3.01

† Standard deviation

In addition, the average difference of neurosis scores was significant between experimental and control groups (p < 0.05). Life skills instruction was effective in decreasing the neurosis of students. The average difference of stress-depression scores between experimental and control group was significant (p < 0.05). Life skills instruction was effective in decreasing the stress-depression of students.

Also, the results show that the difference of vitality average scores was significant between experimental and control groups (p < 0.05), so it is concluded that life skills instruction is effective in increasing the vitality of students. The difference of life determination average score was significant between experimental and control groups (p = 0.05). Life skills instruction was effective in increasing the life determination of the students.

**Table 2:** Effect of test result between subjects

**Source**	**Dependent variables**	**Total square**	**R** ^2^	**df** †	**Average square**	**F**	**Sig.**
**Experimental group**	Subjective well-being	32.65	0.481	1	32.65	25.95	0.000
	Neurosis	04.03	0.297	1	04.03	11.83	0.002
	Stress-depresseion	11.02	0.186	1	02.52	06.40	0.017
	Vitality	06.94	0.231	1	02.08	08.39	0.007
	Life determination	07.28	0.122	1	1	03.87	0.059
**Control group**	Subjective well-being	35.23	0.248	1	01.25		0.430
	Neurosis	00.29	0.457	1	00.34		0.670
	Stress-depression	11.02	0.006	1	00.39		0.260
	Vitality	00.23	0.147	1	00.24		0.080
	Life determination	07.28	0.022	1	00.26		0.450

† Degree of freedom

**Table 3:** Comparison of subjective well-being, neurosis, stress-depression, vitality, and life determination scores between experimental and control groups

**Variable**	**Group**	**M** †	**SD** ‡	**df** §	**t**	**p**
**Subjective well-being**	Experimental	−0.74	1.30	28	−5.09	0.000
	Control	−1.34	0.90			
**Neurosis**	Experimental	−3.28	0.68	28	3.44	0.002
	Control	−4.02	0.46			
**Stress-depression**	Experimental	−2.70	0.69	28	2.53	0.01
	Control	−3.28	0.55			
**Vitality**	Experimental	−3.63	0.55	28	−2.89	0.007
	Control	−3.10	0.43			
**Life determination**	Experimental	−3.18	0.51	28	−1.96	0.05
	Control	−2.82	0.50			

† Mean;

‡ Standard deviation;

§ Degree of freedom

## Discussion

The question of this study was that which studied variables (i.e., SWB, neurosis, stress-depression, vitality, and life determination) were influenced most by training life skills in female high school students. It was found that SWB had the greatest increase in score following training compared to other variables.

The results showed that life skills training is the cause of increase in SWB of female students in post-test stage and score difference observed between experimental and control groups was significant and is in agreement with similar previous studies (14, 15).

SWB points to our thinking and feelings in life, and on the other hand, when we assess ourselves what kind of cognition and feeling we find (16). On the other hand, it is positive assessment of life and balance between positive and negative affect, that positive assessment have high value in acting in the best state during adulthood like having mental health (this means having high satisfaction in life and low psychological pathology) (17).

Vivan et al. pointed to the importance of lifestyle and kind of person’s encountering environment and life problems in person’s SWB grade (18). Creation and strengthening abilities and skill in a bad situation is the cause of person’s mental health. Zollinger et al. in a survey found that life skills training in 3 years course has a key role in forming abilities and changing idea of students (19). Ramesht and Farshad showed that life skill instruction resulted in the growth of student’s mental health (7). Phuphaibul and colleagues showed that after the copped skills instruction, the experimental group paraded better copping behaviors than the control group, and gained higher mental health (20). Matsuda and Uchiyama express that student following copping skills instruction, announced less educational mental pressure and copping skills instruction resulted in the prevention and reduction of mental disorders (12).

In general, it is concluded that, individuals who have lower mental health and also suffer from anxiety, depression and stress, often do not have sufficient information about these disorders and do not know how to cope with them and also do not have sufficient information about skills such as problem solving, feeling expression, setting goals, decision making, planning, identification, registrations the negative thought, and replacing the positive ones.

Life skills are a collection of organized manners that help us to cope with life problems in daily life. These skills are different and are defined in different ways in various cultures. Some of these capacities are trained to children at school or by families. 

Limitations of the study were having no special program for the control group and including only females.

## Authors' contributions

RSM and MK conceived and designed the evaluation. RSM collected the clinical data. HM interpreted the clinical data. RSM performed the statistical analysis and drafted the manuscript. All authors read and approve the final manuscript.

## Footenote

**Declaration of interest:** None.
